# High Immunoproteasome Activity and sXBP1 in Pediatric Precursor B-ALL Predicts Sensitivity towards Proteasome Inhibitors

**DOI:** 10.3390/cells10112853

**Published:** 2021-10-22

**Authors:** Lenka Besse, Andrej Besse, Marianne Kraus, Elmer Maurits, Herman S. Overkleeft, Beat Bornhauser, Jean-Pierre Bourquin, Christoph Driessen

**Affiliations:** 1 Department of Oncology and Hematology, Experimental Oncology and Hematology, Cantonal Hospital St. Gallen, 9007 St. Gallen, Switzerland; Andrej.besse@kssg.ch (A.B.); marianne.kraus@kssg.ch (M.K.); 2 Gorlaeus Laboratories, Leiden Institute of Chemistry, Leiden University, 2333 CD Leiden, The Netherlands; e.maurits@lic.leidenuniv.nl (E.M.); h.s.overkleeft@lic.leidenuniv.nl (H.S.O.); 3 Department of Oncology and Children’s Research Centre, University Children’s Hospital Zurich, 8032 Zürich, Switzerland; Beat.Bornhauser@kispi.uzh.ch (B.B.); Jean-Pierre.Bourquin@kispi.uzh.ch (J.-P.B.)

**Keywords:** BCP-ALL, T-ALL, pediatric leukemia, immunoproteasome, proteasome inhibitors, LU015i, activity-based probes, proteasome activity

## Abstract

Proteasome inhibitors (PIs) are approved backbone treatments in multiple myeloma. More recently, inhibition of proteasome activity with the PI bortezomib has been clinically evaluated as a novel treatment strategy in pediatric acute lymphoblastic leukemia (ALL). However, we lack a marker that could identify ALL patients responding to PI-based therapy. By using a set of activity-based proteasome probes in conjunction with cytotoxicity assays, we show that B-cell precursor ALL (BCP-ALL), in contrast to T-ALL, demonstrates an increased activity of immunoproteasome over constitutive proteasome, which correlates with high ex vivo sensitivity to the PIs bortezomib and ixazomib. The novel selective PI LU015i-targeting immunoproteasome β5i induces cytotoxicity in BCP-ALL containing high β5i activity, confirming immunoproteasome activity as a novel therapeutic target in BCP-ALL. At the same time, cotreatment with β2-selective proteasome inhibitors can sensitize T-ALL to currently available PIs, as well as to β5i selective PI. In addition, levels of total and spliced forms of XBP1 differ between BCP-ALL and T-ALL, and only in BCP-ALL does high-spliced XBP1 correlate with sensitivity to bortezomib. Thus, in BCP-ALL, high immunoproteasome activity may serve as a predictive marker for PI-based treatment options, potentially combined with XBP1 analyses.

## 1. Introduction

Proteasome inhibitors (PIs), such as boronate-based bortezomib and ixazomib and epoxyketone-based carfilzomib, are approved therapeutic backbones in multiple myeloma (MM), a malignancy of terminally differentiated B-cells called plasma cells [1]. Bortezomib has more recently entered clinical evaluation in pediatric relapsed/refractory leukemia [2,3], whereas carfilzomib and ixazomib were tested as promising novel PIs in pediatric leukemia in vitro [4,5]. Proteasomes are large protein complexes with three main catalytic subunits, β1, β2 and β5. The β5 proteasome subunit was initially defined as rate limiting for functional proteasomal degradation and later identified to allosterically activate the β1 subunit [6,7,8], whereas the importance of the β2 subunit as a target for functional co-inhibition, together with β5 with subsequent cytotoxicity, was recently shown in MM and breast cancer [9,10,11]. Inhibition of proteasome disrupts the equilibrium between protein production and disposal of misfolded or nonfunctional proteins, leading to proteotoxic stress; excess activation of the unfolded protein response (UPR), including transcriptional control by inositol-requiring enzyme 1 (IRE1); and splicing of its downstream transcription factor X-box binding protein 1 (XBP1), leading, finally, to cell death [12].

In hematopoietic cells and lymphoid tissue, the standard, constitutive subunits β1, β2, and β5 are replaced by their immune counterparts β1i (LMP2), β2i (MECL1) and β5i (LMP7), respectively, to form the immunoproteasome [13,14]. The immunoproteasome has higher β5i and β2i subunit activity but lower β1i activity than the constitutive proteasome, resulting in the alternative cleavage of proteins into peptides that are presented by major histocompatibility complex (MHC) class I molecules [15]. The importance of the immunoproteasome as a target for interference in pediatric acute lymphoblastic leukemia (ALL) has been suggested earlier, based on increased expression of immunoproteasome subunits, together with functional studies showing promising cytotoxic activity of PIs in this disease [16,17,18]. The recent development of immunoproteasome-specific PIs may, therefore, allow selective targeting of such increased immunoproteasome activity in ALL to overcome drug resistance while sparing the vast majority of tissues not expressing the immunoproteasome, thus considerably reducing toxicity. However, biologically relevant functional immunoproteasome activity may not be directly deduced from immunoproteasome subunit expression analysis, because proteasomes are highly dynamic structures and subjects of multiple post-translational modifications that may activate or inactivate fully assembled proteasomes [19]. To date, we lack data that relate the activity of individual proteolytic subunits of the immunoproteasome versus the constitutive proteasome to the cytotoxic activity of PIs in the two major subsets of pediatric ALL, B-cell precursor ALL (BCP-ALL) and T-lineage ALL.

Terminally differentiated normal and malignant B-cells (plasma cells) show higher levels of activity of the IRE1/XBP1 UPR branch in comparison to other tumors or peripheral blood mononuclear cells [20]. At the same time, normal precursor B-cells and BCP-ALL cells express high XBP1, and its ablation causes cell cycle arrest and apoptosis [21]. Therapeutic inhibition of activated XBP1 has been shown to be cytotoxic in MM and BCP-ALL in vitro [21,22] but so far was not moved further into clinical practice. Moreover, high levels of spliced XBP1 directly correlated with better response to bortezomib in MM [20]. Until now, there have been no data that associate the level of total or spliced XBP1 with a response to bortezomib in BCP-ALL.

Here, we used a unique set of proteasome activity-binding probes (ABPs) [23] that selectively visualize all proteolytically active subunits of the immunoproteasome and the constitutive proteasome in a direct, activity-dependent fashion. Activity patterns of the subunits of the immunoproteasome and the constitutive proteasome were analyzed in a set of patient-derived BCP-ALL and T-ALL xenograft samples and related to PI-induced cytotoxicity. This served to dissect the relationship between the activity of immunoproteasome versus constitutive proteasome subunits and the cytotoxicity of nonselective, first-generation, boronate-based PI bortezomib and ixazomib versus current-generation proteasome inhibitors targeting individual proteolytic subunits (such as β2) or the rate-limiting subunit of the immunoproteasome (β5i). Moreover, we determined levels of total, spliced and unspliced variants of XBP1 in BCP-ALL and T-ALL xenograft samples and correlated them to the cytotoxicity of bortezomib, with the aim to assess if total or spliced XBP1 may serve as a marker of better response to bortezomib in ALL patients.

## 2. Materials and Methods

### 2.1. Patients’ Samples

Patient-derived xenografts of BCP-ALL and T-ALL samples were generated at the University Children’s Hospital Zurich, Zurich, Switzerland. Primary human ALL cells were recovered from cryopreserved bone marrow aspirates of patients enrolled in the ALL-BFM 2000 (Multi-Center Study for the Treatment of Children and Adolescents with Acute Lymphoblastic Leukemia), ALL-BFM 2009 (International Collaborative Treatment Protocol for Children and Adolescents with Acute Lymphoblastic Leukemia) and ALL-REZ-BFM 2002 (Multi-Center Study for Children with Relapsed Acute Lymphoblastic Leukemia) studies, as indicated before [24]. For sample collection, informed consent was given in accordance with the Declaration of Helsinki and the ethics committee of the Kanton Zurich (Approval Number: 2014-0383). The baseline characteristics of patients’ samples are included in Table 1. Generation of patient-derived xenografts (PDXs) used in the study was described previously [24]. Briefly, 1 × 10^5^ to 5 × 10^6^ of viable primary ALL cells were intrafemorally injected in NSG mice. Leukemia progression was monitored in peripheral blood by flow cytometry using mouse-specific anti-CD45 and human-specific anti-CD45 and anti-CD19 or anti-CD7 antibodies. Xenograft identity was verified by DNA fingerprinting using the commercial AmpFLSTR NGM SElect polymerase chain reaction amplification kit (Thermo Fisher Scientific, Waltham, MA, USA).

### 2.2. Chemicals

Bortezomib (#S1013) and ixazomib (#S2181) were obtained from commercial sources (Selleck Chemicals, Houston, TX, USA). LU015i, LU102 and ABP were synthesized at Leiden University. Detailed information about the proteasome inhibitors used in the study is presented in Table S1.

### 2.3. Activity-Based Proteasome Probes Profiling and Calculation of Activity Ratios

Activity of proteasome subunits was assessed on a protein lysate by SDS-PAGE after 1 h/37 °C incubation with the set of subunit-selective activity-based probes (ABPs) that differentially visualize individual activities of β1, β2 and β5 subunits of the constitutive and immunoproteasome, as described [23]. Protein subunits were separated by SDS-PAGE, and gel images were acquired using the Fusion Solo S Western Blot and Chemi Imaging System (Vilber Lourmat, Collegien, France). Quantification of the activity was performed using Fiji (open-source image-processing package based on ImageJ) [25]. For each sample, the ratio of activity of the immunoproteasome vs. constitutive proteasome subunits was calculated by dividing the band intensity of each of the immunoproteasome subunits by the band intensity of the corresponding constitutive proteasome subunit.

### 2.4. Western Blotting and Calculation of Protein Expression Ratios

After SDS-PAGE of ABP-labeled samples, separated proteins were blotted from a gel on a PVDF membrane, blocked with RotiBlock for 1 h (Carl Roth, Karlsruhe, Germany) and incubated overnight with anti-β5c and anti-β5i (#PW8895 and #PW8355; Enzo Life Sciences, Farmingdale, NY, USA) and anti-GAPDH antibody (#G8795, Sigma-Aldrich, Buchs, Switzerland) that served as a loading control. β5c and β5i protein levels for each sample were quantified using Fiji software. Normalization of each sample was performed to GAPDH as a loading control and to β5c and β5i protein levels expressed in peripheral blood mononuclear cells that served as an internal control between the individual gels/membranes.

### 2.5. CTG Cytotoxicity Assay and Data Normalization

Briefly, 1 × 10^4^ cells per well were seeded in a white, flat-bottom, 96-well plate (Corning Switzerland, Root, Switzerland). The cells were exposed to increasing doses of proteasome inhibitors in 100 µL of media per well for 48 h, and cell viability was determined using the CellTiter-Glo luminescent cell viability assay (Promega, Madison, WI, USA) according to the manufacturer’s protocol. The cytotoxicity of the drugs was normalized to control, untreated cells, and for each chemical and sample, a dose–response curve was generated.

### 2.6. RNA Extraction, qRT-PCR and Data Normalization

Total RNA was extracted using the Direct-Zol RNA kit (Zymo Research, Irvine, CA, USA), cDNA was reverse transcribed using the High-Capacity cDNA Reverse Transcription Kit (Thermo Fisher Scientific, Waltham, MA, USA), and expression of spliced (sXBP1), unspliced (uXBP1) and total (tXBP1) XBP1 was determined using the SYBR Green method and previously described primers [26] and normalized to GAPDH as a housekeeping gene. Next, the GAPDH-normalized sXBP1 and uXBP1 expression was used to determine the s/u XBP1 expression in each sample.

### 2.7. Statistical Analysis

Dose–response curves were generated using a nonlinear fit. The IC_50_ value of each chemical was determined using nonlinear regression analysis of dose–response curves. The Mann–Whitney U test was used for the comparison of the BCP-ALL and T-ALL groups. For paired analysis, Wilcoxon’s paired test was used. Correlation coefficients between the activity ratios of β5i/c, β1i/c and β2i/c or between the XBP1 s/u and the cytotoxicity of proteasome inhibitors were calculated using Spearman’s rank correlation. Values of *p* < 0.05 were considered statistically significant. Statistical evaluation was performed in GraphPad Prism v5 (GraphPad Software, La Jolla, CA, USA).

## 3. Results

### 3.1. BCP-ALL, but Not T-ALL Show Increased Immunoproteasome over Constitutive Proteasome Activity

**Figure 1:**
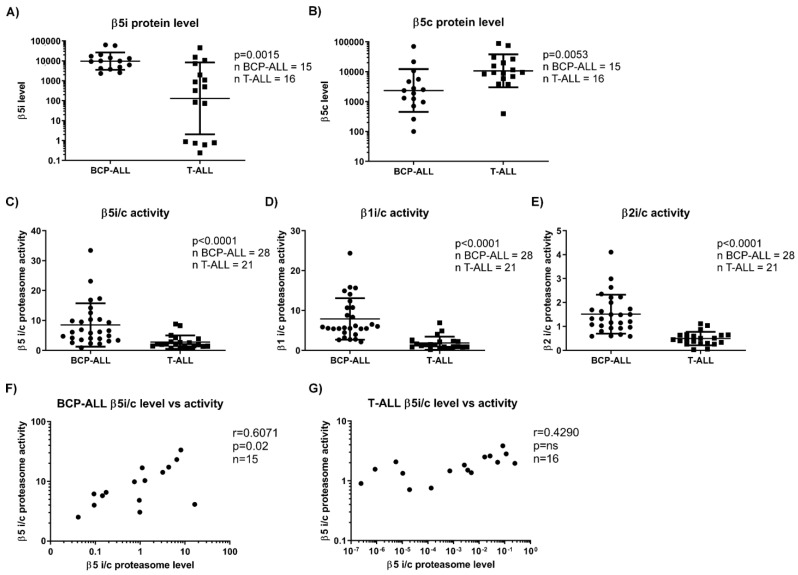
Expression levels and activity of the immunoproteasome vs. constitutive proteasome in BCP-ALL and T-ALL. (**A**) Comparison of levels of the constitutive proteasome β5 subunit (β5c) between BCP-ALL and T-ALL samples. Data represent geometric mean ± SD. *p* was obtained with Mann–Whitney U test. (**B**) Comparison of levels of the immunoproteasome β5 subunit (β5i) between BCP-ALL and T-ALL samples. Data represent geometric mean ± SD. *p* was obtained with Mann–Whitney U test. (**C**) Comparison between the ratio of activity of proteasome β5i versus β5c. Data represent mean ± SD. *p* was obtained with Mann–Whitney U test. (**D**) Comparison between the ratio of activity of proteasome β1i versus β1c. Data represent mean ± SD. *p* was obtained with Mann–Whitney U test. (**E**) Comparison between the ratio of activity of proteasome β2i versus β2c. Data represent mean ± SD. *p* was obtained with Mann–Whitney U test. (**F**) Correlation between the ratio of activity of proteasome β5i versus β5c subunits and the ratio of expression levels of proteasome β5i versus β5c subunits in BCP-ALL samples. r and *p* were obtained with Spearman’s correlation. (**G**) Correlation between the ratio of activity of proteasome β5i versus β5c subunits and the ratio of expression levels of proteasome β5i versus β5c subunits in T-ALL samples. r and *p* were obtained with Spearman’s correlation. BCP-ALL = B-cell precursor acute lymphoblastic leukemia, T-ALL = T-lineage acute lymphoblastic leukemia, i = immunoproteasome, c = constitutive proteasome.

Initially, 15 BCP-ALL and 16 T-ALL samples were compared with respect to the expression levels of the constitutive β5c and immunoproteasome β5i subunits. The data confirmed previous observations indicating that BCP-ALL samples exhibit significantly increased expression levels of the β5i subunit and decreased levels of the β5c subunit compared to T-ALL (Figure 1A,B) [17]. Since such an antibody-dependent approach indiscriminately detects both the active and inactive forms of the proteasome subunits, differences in protein expression levels of individual proteasome subunits do not allow deducing the differential activity of such subunits within the fully assembled proteasome multiprotein complex. Therefore, we used ABP labeling in a larger set of 28 BCP-ALL and 21 T-ALL samples (detailed characteristics provided in Table 1) to directly assess the activity of the individual proteolytic proteasome and immunoproteasome β subunits, which we present as the activity ratio between the constitutive and the corresponding immunoproteasome subunits [23].

In line with the increased expression levels of immunoproteasome subunits in BCP-ALL samples, our data showed a significantly increased activity ratio of the immune vs. constitutive proteasome (βi/c) for all subunits, in contrast to T-ALL (Figure 1C–E). While βi/c activity ratios for the proteasome β1, β2 and β5 subunits varied considerably for individual samples of BCP-ALL and T-ALL, the mean ratio of βi/c activity was about three times higher in BCP-ALL compared to T-ALL (Table S2). In BCP-ALL, the activity ratio between β5i/c correlated with the protein expression ratio, whereas in T-ALL, no such correlation was found (Figure 1F,G). Thus, pediatric BCP-ALL cells, but not T-ALL, show increased activity in all proteolytically active immunoproteasome subunits, which correlates with protein expression level.

### 3.2. Cytotoxicity of Bortezomib and Ixazomib Correlates with Proteasome Activity Ratios in BCP-ALL, but Not in T-ALL

**Figure 2:**
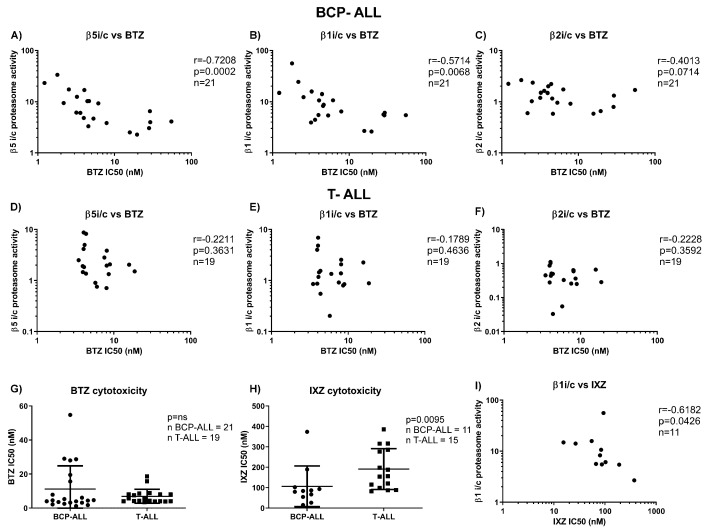
Correlation between the BCP-ALL and T-ALL samples and the cytotoxicity of bortezomib and ixazomib. Correlation between the ratio of activity of proteasome β5i versus β5c (**A**), β1i versus β1c (**B**) and β2i versus β2c (**C**) proteasome subunits, determined by ABPs, and cytotoxicity of BTZ in BCP-ALL, expressed as individual IC_50_ values. r and *p* were obtained with Spearman’s correlation. Correlation between the ratio of activity of proteasome β5i versus β5c (**D**), β1i versus β1c (**E**) and β2i versus β2c (**F**) subunits, determined by ABPs, and cytotoxicity of BTZ in T-ALL, expressed as individual IC_50_ values. r and *p* were obtained with Spearman’s correlation. (**G**) Comparison of the IC_50_ values for BTZ between BCP-ALL and T-ALL samples. *p* was obtained with Mann–Whitney U test. (**H**) Comparison of the IC_50_ values for IXZ between BCP-ALL and T-ALL samples, *p* was obtained with Mann–Whitney U test. (**I**) Correlation between the ratio of activity of proteasome β1i versus β1c proteasome subunits, determined by ABPs, and cytotoxicity of IXZ in BCP-ALL, expressed as individual IC_50_ values. r and *p* were obtained with Spearman’s correlation. BTZ = bortezomib, IXZ = ixazomib, BCP-ALL = B-cell precursor acute lymphoblastic leukemia, T-ALL = T-lineage acute lymphoblastic leukemia, i = immunoproteasome, c = constitutive proteasome.

Next, we correlated the different βi/c activity ratios for BCP-ALL and T-ALL to the ex vivo cytotoxicity of bortezomib and ixazomib in a larger cohort of BCP-ALL and T-ALL patient samples. Due to the limited amount of material from individual xenografts, we were able to test 21 BCP-ALL and 19 T-ALL samples for bortezomib cytotoxicity, but only 11 BCP-ALL and 15 T-ALL samples for ixazomib cytotoxicity, and relate them to the proteasome activity ratios. In BCP-ALL, activity ratios for β5i/c and β1i/c inversely correlated with the IC_50_ values of bortezomib (Figure 2A–C), consistent with the observation that bortezomib at higher concentrations co-inhibits both constitutive and immunoproteasome β1 subunits in addition to the β5 subunits [9], and it may be more selective towards the immunoproteasome subunits [27,28]. However, no such correlation between activity ratios and bortezomib-induced cytotoxicity was observed in T-ALL samples (Figure 2D–F). There was no significant difference between unselected BCP-ALL and T-ALL samples in regard to bortezomib cytotoxicity (Figure 2G). On the contrary, BCP-ALL samples were significantly more sensitive to ixazomib ex vivo compared to T-ALL (Figure 2H), and ixazomib IC_50_ values correlated with β1i/c ratios only in BCP-ALL (Figure 2I). Ixazomib, like bortezomib, co-inhibits the β5 and β1 subunits of the constitutive and immunoproteasome, with high selectivity towards the β1 or β1i subunit [5,9]. Our findings demonstrate a positive correlation between the high activity of immunoproteasome subunits and high sensitivity to bortezomib and ixazomib for BCP-ALL, in contrast to T-ALL.

### 3.3. The β5 Immunoproteasome-Selective Inhibitor LU-015i Is Cytotoxic in BCP-ALL, and Its Cytotoxicity Correlates with β5i/c Activity Ratio

**Figure 3:**
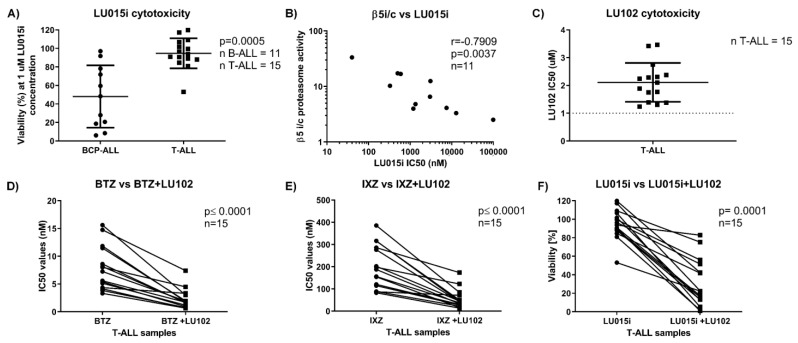
Cytotoxicity of subunit-selective proteasome inhibitors in BCP-ALL and T-ALL and correlation with immunoproteasome activity. (**A**) Comparison of the cytotoxicity of LU015i between BCP-ALL and T-ALL samples, *p* was obtained with Mann–Whitney U test. (**B**) Correlation between the ratio of activity of proteasome β5i versus β5c, determined by ABPs, and cytotoxicity of LU015i in BCP-ALL, expressed as individual IC_50_ values. r and *p* were obtained with Spearman’s correlation. (**C**) IC_50_ values of LU102 in T-ALL samples. (**D**) Paired comparison of IC_50_ values of BTZ alone or combined with fixed dose of LU102 (1 µM) determined 48 h after the continuous treatment in T-ALL. *p* was obtained with Wilcoxon’s paired test. (**E**) Paired comparison of IC_50_ values of IXZ alone or combined with fixed dose of LU102 (1 µM) determined 48 h after the continuous treatment in T-ALL. *p* was obtained with Wilcoxon’s paired test. (**F**) Cell viability (normalized to untreated control cells as 100%) in T-ALL after 48 h treatment with 1 µM LU015i alone or in combination with 1 µM LU102. *p* was obtained with Wilcoxon’s paired test. BTZ = bortezomib, IXZ = ixazomib, LU102 = a β2 selective inhibitor, LU015i = a β5i selective inhibitor, BCP-ALL = B-cell precursor acute lymphoblastic leukemia, T-ALL = T-lineage acute lymphoblastic leukemia, i = immunoproteasome, c = constitutive proteasome.

The increased activity of β5i over β5c in BCP-ALL led us to test LU015i, a recently developed β5i selective inhibitor [29], with respect to potential antileukemic activity in pediatric ALL. We assessed its cytotoxicity in the above-mentioned set of 11 BCP-ALL and 15 T-ALL samples. Although we performed the dose–response experiments, in most T-ALL samples the IC_50_ values were not reached or were reached at a high dose of the inhibitor, where it is not selective anymore for the β5i subunit. Therefore, we present data where we compared the cytotoxicity of LU015i at a 1 µM dose, where the inhibitor is selective for the β5i subunit. BCP-ALL cells showed significantly higher sensitivity to LU015i compared to T-ALL (Figure 3A), likely reflecting the higher ratio of β5 immunoproteasome versus constitutive proteasome activity in BCP-ALL samples compared to T-ALL. Consequently, the cytotoxicity of LU015i strongly correlated with the β5i/c activity ratio in BCP-ALL (Figure 3B), but not with the β5i/c expression level ratio. In summary, high activity of the proteasome β5i subunit may be used as a potential therapeutic target for next-generation immunoproteasome-specific inhibitors, like the β5 immunoproteasome-selective inhibitor LU015i, in BCP-ALL.

### 3.4. The β2 Proteasome-Selective Inhibitor LU-102 Potentiates the Activity of Clinically Available Proteasome Inhibitors in T-ALL

LU102, the β2c and β2i selective inhibitor, sensitizes MM cells to bortezomib and to immunoproteasome inhibitors [11,30,31]. We therefore assessed the cytotoxic activity of LU102 in T-ALL and tested if co-inhibition of β2 proteasome activity with the β5-targeting inhibitors bortezomib and ixazomib and the β5i inhibitor LU015i may be more cytotoxic. Single-agent LU102 at a concentration of 1 µM, which eliminates β2 activity with high selectivity [9], was not cytotoxic in T-ALL samples (Figure 3C). Combination treatment with LU102 potentiated the cytotoxic activity of bortezomib, ixazomib and LU015i in all tested T-ALL samples (Figure 3D–F). Thus, co-inhibition of the proteasome β2 subunit sensitizes T-ALL samples to currently approved and novel, next-generation proteasome inhibitors.

### 3.5. BCP-ALL Keep Spliced XBP1, Which Correlates with Sensitivity to Bortezomib

**Figure 4:**
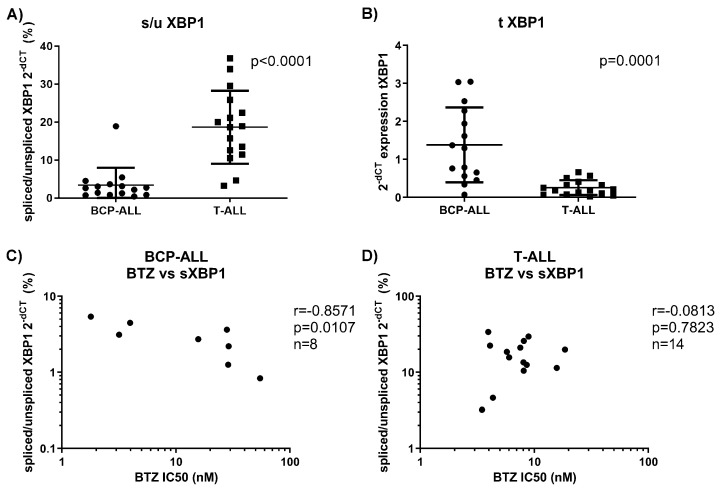
XBP1 transcripts in BCP-ALL and T-ALL. (**A**) s/uXBP1 determined by qRT-PCR in BCP-ALL and T-ALL. Data represent mean ± SD. *p* was obtained with Mann–Whitney U test. (**B**) Total variant of XBP1 determined by qRT-PCR. Data represent mean ± SD. *p* was obtained with Mann–Whitney U test. (**C**) Correlation between s/u XBP1 ratio and BTZ cytotoxicity in BCP-ALL. r and *p* were obtained with Spearman’s correlation. (**D**) Correlation between s/u XBP1 ratio and BTZ cytotoxicity in T-ALL. r and *p* were obtained with Spearman’s correlation. BTZ = bortezomib, s/uXBP1 = ratio of spliced vs. unspliced variants of XBP1, tXBP1 = total variant of XBP1 transcript, qRT-PCR = quantitative real-time PCR, BCP-ALL = B-cell precursor acute lymphoblastic leukemia, T-ALL = T-lineage acute lymphoblastic leukemia.

We evaluated XBP1 (total variant and variants indicating high ER stress: spliced vs. unspliced XBP1; s/u) in a small cohort of 8 BCP-ALL and 14 T-ALL samples, for which we had enough material to relate the results to the selective sensitivity to proteasome inhibitors observed above. BCP-ALL samples showed significantly higher levels of total XBP1 compared to T-ALL (Figure 4A), which is consistent with previous findings of high XBP1 expression in BCP-ALL [21]. At the same time, BCP-ALL showed a lower ratio of s/u XBP1 expression compared to T-ALL (Figure 4B), but only in BCP-ALL did the s/u XBP1 expression ratio correlate negatively with cytotoxicity to bortezomib (Figure 4C,D). We did not observe any correlation between total XBP1 expression and bortezomib cytotoxicity in both of the cohorts. Therefore, only in BCP-ALL is a high expression of spliced XBP1 associated with increased sensitivity to bortezomib treatment.

## 4. Discussion

The present study demonstrates increased activity of the immunoproteasome over the constitutive proteasome, which correlates with high ex vivo sensitivity to the PIs bortezomib and ixazomib in xenografts obtained from primary patient samples of pediatric BCP-ALL. Previous work did not find a correlation between βi/c protein levels and bortezomib sensitivity in ALL, although ALL samples were more sensitive to bortezomib than AML [16]. At the same time, ALL samples were equally sensitive to ixazomib as AML samples [5]. Our work extends published data and shows that the correlation between sensitivity to bortezomib or ixazomib, and the immunoproteasome to constitutive proteasome activity ratio is present solely in BCP-ALL, but not in T-ALL samples. Moreover, instead of measuring proteasome subunit protein levels, we assessed the activity of each of the active proteasome subunits using ABPs. ABPs are developed based on the covalent binding of small inhibitors with active site residues of catalytic subunits. They have a strong preference for a specific subunit type, and thus, they are able to directly visualize the availability and reactivity of the active proteasomes rather than their abundance in a limited amount of samples of primary cells [32]. In this respect, the use of ABPs for assessment of proteasome activity in patient-derived primary cell samples seems a feasible approach to identify potential responders to PI-based therapy.

We clearly show that increased immunoproteasome activity exclusively sensitizes BCP-ALL cells to immunoproteasome inhibitors, confirming immunoproteasome activity as a novel therapeutic target in BCP-ALL. In our work, we used a novel selective β5i inhibitor LU015i. Compared to the previously developed β5i inhibitor PR957 (ONX0914), which has already been tested in ALL [16] and whose cytotoxicity did not correlate with β5i expression levels, LU015i is more selective towards β5i subunit in a broader range of concentrations [23], and its cytotoxicity correlates strongly with the β5i activity, as we show here.

Unlike BCP-ALL, T-ALL cells show balanced activity of both types of proteasomes. We assume that it is likely due to this fact they do not respond to the immunoproteasome-selective inhibitor by induction of cytotoxicity, because the preserved activity of the constitutive proteasome is likely sufficient to ensure cell survival during immunoproteasome inhibition, as we have previously shown [9]. Co-targeting of β2 activity is a promising strategy to sensitize MM cells to immunoproteasome inhibitors [31] and to overcome intrinsic or acquired PI resistance to β5-directed proteasome inhibitors in MM and solid tumors [10,11]. Likewise, co-inhibition of β2 proteasome activity sensitized T-ALL cells to immunoproteasome-selective inhibitors, as well as to bortezomib and ixazomib. Our data provide a strong rationale for the use of a combination therapy that includes immunoproteasomes and β2 inhibitors in T-ALL.

XBP1 expression governs late events in plasma cell differentiation and thus is required for normal plasma cell development and function [33]. High XBP1 expression and presence of its spliced and more active form, sXBP1, is a hallmark of MM [34]. At the same time, XBP1 expression is high in BCP-ALL and peaks during normal early B-cell development at the pre-B-cell receptor checkpoint in human bone marrow, where it is critical for proliferation and survival of the cells [21]. Clinically, high total XBP1 levels are indicative of good clinical response to bortezomib treatment in MM, although no correlation between XBP1 RNA expression and response to therapy was found [35]. At the same time, high levels of the sXBP1 variant were associated with bortezomib sensitivity in MM patients [20]. Our data show that BCP-ALL cells have higher expression of total XBP1 in comparison to T-ALL cells, which supports previous data, where BCP-ALL cells showed high XBP1 expression [21]. On the contrary, we show that the level of activated sXBP1 in our cohort of BCP-ALL is rather low in comparison to T-ALL. Nevertheless, the ratio between spliced and unspliced forms of XBP1 is associated with sensitivity to bortezomib only in the BCP-ALL cohort of patients, suggesting that spliced XBP1 may be indicative of bortezomib response in BCP-ALL. Previously, it has been shown that high total XBP1 levels at diagnosis correlated with poor outcome in a clinical trial of high-risk childhood ALL patients treated with conventional chemotherapy (COG P9906) [21], suggesting that high XBP1 is a marker of aggressive disease. Our data suggest that high sXBP1 expression in BCP-ALL may be a marker of better response to bortezomib-containing therapy and provide a rationale to use bortezomib in patients with high sXBP1 expression.

We acknowledge the limitation of our study, which is the low number of patients used in the analysis. Thus, further studies will need to determine if high sXBP1 could be used as a predictive marker for bortezomib sensitivity in pediatric patients with BCP-ALL who may not be responding to initial, standard treatment protocols with high-dose chemotherapy and glucocorticoids.

## 5. Conclusions

In conclusion, this work provides a strong rationale for the development of immunoproteasome inhibitors in the next line of treatment of patients with refractory BCP-ALL. Current-generation selective PI targeting β5i has the potential to induce cytotoxicity in BCP-ALL containing high β5i activity, with the potential for very high target selectivity and thus low toxicity due to the lack of immunoproteasome activity in cells of non-antigen-presenting origin. In addition, cotreatment with β2-selective proteasome inhibitors can sensitize T-ALL to currently available PIs, as well as to β5i selective PI. Finally, immunoproteasome activity levels can be determined by ABP in pediatric BCP-ALL, which represents an attractive strategy for use as a predictive marker for proteasome inhibitor-based treatment options, potentially combined with sXBP1 analyses, in particular in pediatric BCP-ALL.

## Figures and Tables

**Table 1:** Characteristics of patients included in the study, for which proteasome activity was assessed by activity-based probes profiling. MLL-AF4: chimeric protein of mixed-lineage leukemia (MLL)-AF4, TCF3-PBX1: fusion protein of transcription factor 3 and PBX homeobox 1, HHD: high hyperdiploidy, AML1: acute myeloid leukemia 1. * According to ALL-BFM 2000 risk stratification criteria.

	BCP-ALL	T-ALL
No. of patients	28	21
Sex: male/female (%)	9 (32%)–19 (68%)	18 (86%)–3 (14%)
Age (median; min–max)	9.7 (0.3–17.6)	5 (2–18.8)
Sample characteristics		
Diagnosis (%)	22 (78.6%)	17 (81%)
Relapse (%)	6 (21.4%)	4 (19%)
Risk at diagnosis (%) *		
Standard risk	9 (40.9%)	1 (5.9%)
Medium risk	0	7 (41.2%)
High risk	5 (22.7%)	6 (35.3%)
Very high risk	8 (36.4%)	3 (17.6%)
Cytogenetic aberrations in BCP-ALL		
MLL-AF4_t (4;11)	5	
TCF3-PBX1_t (1;19)	5	
HHD	3	
AML1 amplification	1	
Immunophenotype in T-ALL (%)		
Cortical		10 (47.6%)
Mature		7 (33.3%)
Pre		4 (19.1%)

## Supplementary Materials

The following are available online at https://www.mdpi.com/article/10.3390/cells10112853/s1, Table S1: Detailed characteristics of proteasome inhibitors used in the study, Table S2: Activity of immunoproteasome versus constitutive proteasome subunits (i/c) in BCP-ALL and T-ALL.

## References

[1] Moreau P., Richardson P.G., Cavo M., Orlowski R.Z., San Miguel J.F., Palumbo A., Harousseau J.L. (2012). Proteasome inhibitors in multiple myeloma: 10 years later. Blood.

[2] Horton T.M., Perentesis J.P., Gamis A.S., Alonzo T.A., Gerbing R.B., Ballard J., Adlard K., Howard D.S., Smith F.O., Jenkins G. (2014). A Phase 2 study of bortezomib combined with either idarubicin/cytarabine or cytarabine/etoposide in children with relapsed, refractory or secondary acute myeloid leukemia: A report from the Children’s Oncology Group. Pediatric Blood Cancer.

[3] Messinger Y.H., Gaynon P.S., Sposto R., van der Giessen J., Eckroth E., Malvar J., Bostrom B.C., Therapeutic Advances in Childhood L., Lymphoma C. (2012). Bortezomib with chemotherapy is highly active in advanced B-precursor acute lymphoblastic leukemia: Therapeutic Advances in Childhood Leukemia & Lymphoma (TACL) Study. Blood.

[4] Takahashi K., Inukai T., Imamura T., Yano M., Tomoyasu C., Lucas D.M., Nemoto A., Sato H., Huang M., Abe M. (2017). Anti-leukemic activity of bortezomib and carfilzomib on B-cell precursor ALL cell lines. PLoS ONE.

[5] Roeten M.S.F., van Meerloo J., Kwidama Z.J., Ter Huizen G., Segerink W.H., Zweegman S., Kaspers G.J.L., Jansen G., Cloos J. (2021). Pre-Clinical Evaluation of the Proteasome Inhibitor Ixazomib against Bortezomib-Resistant Leukemia Cells and Primary Acute Leukemia Cells. Cells.

[6] Heinemeyer W., Fischer M., Krimmer T., Stachon U., Wolf D.H. (1997). The active sites of the eukaryotic 20 S proteasome and their involvement in subunit precursor processing. J. Biol. Chem..

[7] Groll M., Heinemeyer W., Jager S., Ullrich T., Bochtler M., Wolf D.H., Huber R. (1999). The catalytic sites of 20S proteasomes and their role in subunit maturation: A mutational and crystallographic study. Proc. Natl. Acad. Sci. USA.

[8] Kisselev A.F., Akopian T.N., Castillo V., Goldberg A.L. (1999). Proteasome active sites allosterically regulate each other, suggesting a cyclical bite-chew mechanism for protein breakdown. Mol. Cell.

[9] Besse A., Besse L., Kraus M., Mendez-Lopez M., Bader J., Xin B.T., de Bruin G., Maurits E., Overkleeft H.S., Driessen C. (2019). Proteasome Inhibition in Multiple Myeloma: Head-to-Head Comparison of Currently Available Proteasome Inhibitors. Cell Chem. Biol..

[10] Weyburne E.S., Wilkins O.M., Sha Z., Williams D.A., Pletnev A.A., de Bruin G., Overkleeft H.S., Goldberg A.L., Cole M.D., Kisselev A.F. (2017). Inhibition of the Proteasome beta2 Site Sensitizes Triple-Negative Breast Cancer Cells to beta5 Inhibitors and Suppresses Nrf1 Activation. Cell Chem. Biol..

[11] Kraus M., Bader J., Geurink P.P., Weyburne E.S., Mirabella A.C., Silzle T., Shabaneh T.B., van der Linden W.A., de Bruin G., Haile S.R. (2015). The novel beta2-selective proteasome inhibitor LU-102 synergizes with bortezomib and carfilzomib to overcome proteasome inhibitor resistance of myeloma cells. Haematologica.

[12] Obeng E.A., Carlson L.M., Gutman D.M., Harrington W.J., Lee K.P., Boise L.H. (2006). Proteasome inhibitors induce a terminal unfolded protein response in multiple myeloma cells. Blood.

[13] Murata S., Takahama Y., Kasahara M., Tanaka K. (2018). The immunoproteasome and thymoproteasome: Functions, evolution and human disease. Nat. Immunol..

[14] Sijts E.J., Kloetzel P.M. (2011). The role of the proteasome in the generation of MHC class I ligands and immune responses. Cell. Mol. Life Sci..

[15] Gaczynska M., Rock K.L., Goldberg A.L. (1993). Gamma-interferon and expression of MHC genes regulate peptide hydrolysis by proteasomes. Nature.

[16] Niewerth D., Franke N.E., Jansen G., Assaraf Y.G., van Meerloo J., Kirk C.J., Degenhardt J., Anderl J., Schimmer A.D., Zweegman S. (2013). Higher ratio immune versus constitutive proteasome level as novel indicator of sensitivity of pediatric acute leukemia cells to proteasome inhibitors. Haematologica.

[17] Niewerth D., Kaspers G.J., Jansen G., van Meerloo J., Zweegman S., Jenkins G., Whitlock J.A., Hunger S.P., Lu X., Alonzo T.A. (2016). Proteasome subunit expression analysis and chemosensitivity in relapsed paediatric acute leukaemia patients receiving bortezomib-containing chemotherapy. J. Hematol. Oncol..

[18] Swift L., Jayanthan A., Ruan Y., Anderson R., Boklan J., Trippett T., Narendran A. (2018). Targeting the Proteasome in Refractory Pediatric Leukemia Cells: Characterization of Effective Cytotoxicity of Carfilzomib. Target. Oncol..

[19] Kors S., Geijtenbeek K., Reits E., Schipper-Krom S. (2019). Regulation of Proteasome Activity by (Post-)transcriptional Mechanisms. Front. Mol. Biosci..

[20] Borjan B., Kern J., Steiner N., Gunsilius E., Wolf D., Untergasser G. (2019). Spliced XBP1 Levels Determine Sensitivity of Multiple Myeloma Cells to Proteasome Inhibitor Bortezomib Independent of the Unfolded Protein Response Mediator GRP78. Front. Oncol..

[21] Kharabi Masouleh B., Geng H., Hurtz C., Chan L.N., Logan A.C., Chang M.S., Huang C., Swaminathan S., Sun H., Paietta E. (2014). Mechanistic rationale for targeting the unfolded protein response in pre-B acute lymphoblastic leukemia. Proc. Natl. Acad. Sci. USA.

[22] Harnoss J.M., Le Thomas A., Shemorry A., Marsters S.A., Lawrence D.A., Lu M., Chen Y.A., Qing J., Totpal K., Kan D. (2019). Disruption of IRE1alpha through its kinase domain attenuates multiple myeloma. Proc. Natl. Acad. Sci. USA.

[23] De Bruin G., Xin B.T., Kraus M., van der Stelt M., van der Marel G.A., Kisselev A.F., Driessen C., Florea B.I., Overkleeft H.S. (2016). A Set of Activity-Based Probes to Visualize Human (Immuno)proteasome Activities. Angew. Chem. Int. Ed. Engl..

[24] Frismantas V., Dobay M.P., Rinaldi A., Tchinda J., Dunn S.H., Kunz J., Richter-Pechanska P., Marovca B., Pail O., Jenni S. (2017). Ex vivo drug response profiling detects recurrent sensitivity patterns in drug-resistant acute lymphoblastic leukemia. Blood.

[25] Schindelin J., Arganda-Carreras I., Frise E., Kaynig V., Longair M., Pietzsch T., Preibisch S., Rueden C., Saalfeld S., Schmid B. (2012). Fiji: An open-source platform for biological-image analysis. Nat. Methods.

[26] Oslowski C.M., Urano F. (2011). Measuring ER stress and the unfolded protein response using mammalian tissue culture system. Methods Enzymol..

[27] Kuhn D.J., Orlowski R.Z. (2012). The immunoproteasome as a target in hematologic malignancies. Semin. Hematol..

[28] Kuhn D.J., Hunsucker S.A., Chen Q., Voorhees P.M., Orlowski M., Orlowski R.Z. (2009). Targeted inhibition of the immunoproteasome is a potent strategy against models of multiple myeloma that overcomes resistance to conventional drugs and nonspecific proteasome inhibitors. Blood.

[29] De Bruin G., Huber E.M., Xin B.T., van Rooden E.J., Al-Ayed K., Kim K.B., Kisselev A.F., Driessen C., van der Stelt M., van der Marel G.A. (2014). Structure-based design of beta1i or beta5i specific inhibitors of human immunoproteasomes. J. Med. Chem..

[30] Geurink P.P., van der Linden W.A., Mirabella A.C., Gallastegui N., de Bruin G., Blom A.E., Voges M.J., Mock E.D., Florea B.I., van der Marel G.A. (2013). Incorporation of non-natural amino acids improves cell permeability and potency of specific inhibitors of proteasome trypsin-like sites. J. Med. Chem..

[31] Downey-Kopyscinski S., Daily E.W., Gautier M., Bhatt A., Florea B.I., Mitsiades C.S., Richardson P.G., Driessen C., Overkleeft H.S., Kisselev A.F. (2018). An inhibitor of proteasome beta2 sites sensitizes myeloma cells to immunoproteasome inhibitors. Blood Adv..

[32] Gan J., Leestemaker Y., Sapmaz A., Ovaa H. (2019). Highlighting the Proteasome: Using Fluorescence to Visualize Proteasome Activity and Distribution. Front. Mol. Biosci..

[33] Reimold A.M., Iwakoshi N.N., Manis J., Vallabhajosyula P., Szomolanyi-Tsuda E., Gravallese E.M., Friend D., Grusby M.J., Alt F., Glimcher L.H. (2001). Plasma cell differentiation requires the transcription factor XBP-1. Nature.

[34] Maestre L., Tooze R., Canamero M., Montes-Moreno S., Ramos R., Doody G., Boll M., Barrans S., Baena S., Piris M.A. (2009). Expression pattern of XBP1(S) in human B-cell lymphomas. Haematologica.

[35] Gambella M., Rocci A., Passera R., Gay F., Omede P., Crippa C., Corradini P., Romano A., Rossi D., Ladetto M. (2014). High XBP1 expression is a marker of better outcome in multiple myeloma patients treated with bortezomib. Haematologica.

