# Supplementary material for: High Immunoproteasome Activity and sXBP1 in Pediatric Precursor B-ALL Predicts Sensitivity towards Proteasome Inhibitors
Source: Cells. 2021 Oct 22;10(11):2853. doi: 10.3390/cells10112853 (PMC8616377; doi:10.3390/cells10112853)
Supplement: Supplementary file 1 [file cells-10-02853-s001.zip › cells-1421183-supplementary.pdf]

Table S1: Detailed characteristics of proteasome inhibitors used in the study.

i.v. = intravenous infusion, s.c. = subcutaneous administration

Proteasome inhibitor	Chemical entity	Route of administration	Clinical Status	Target in the proteasome complex
**Bortezomib**	boronate	i.v., s.c.	Approved for MM	β5 (in higher dose β1), both constitutive and immunoproteasome [1]
**Ixazomib**	boronate	oral	Approved for MM	β5 and β1, both constitutive and immunoproteasome [1]
**LU102**	vinylsulfone	i.v.	preclinical	β2, both constitutive and immunoproteasome [2]
**LU015i**	epoxyketone	i.v.	preclinical	β5 immunoproteasome [3]

References

1. Besse, A.; Besse, L.; Kraus, M.; Mendez-Lopez, M.; Bader, J.; Xin, B.T.; de Bruin, G.; Maurits, E.; Overkleeft, H.S.; Driessen, C. Proteasome Inhibition in Multiple Myeloma: Head-to-Head Comparison of Currently Available Proteasome Inhibitors. *Cell Chem Biol* **2019**, *26*, 340-351 e343, doi:10.1016/j.chembiol.2018.11.007.
2. Geurink, P.P.; van der Linden, W.A.; Mirabella, A.C.; Gallastegui, N.; de Bruin, G.; Blom, A.E.; Voges, M.J.; Mock, E.D.; Florea, B.I.; van der Marel, G.A., et al. Incorporation of non-natural amino acids improves cell permeability and potency of specific inhibitors of proteasome trypsin-like sites. *J Med Chem* **2013**, *56*, 1262-1275, doi:10.1021/jm3016987.
3. de Bruin, G.; Huber, E.M.; Xin, B.T.; van Rooden, E.J.; Al-Ayed, K.; Kim, K.B.; Kisselev, A.F.; Driessen, C.; van der Stelt, M.; van der Marel, G.A., et al. Structure-based design of beta1i or beta5i specific inhibitors of human immunoproteasomes. *J Med Chem* **2014**, *57*, 6197-6209, doi:10.1021/jm500716s.

Table S2: Activity of immunoproteasome versus constitutive proteasome subunits (i/c) in BCP-ALL and T-ALL. The individual activity of each of the active proteasome β subunit (β1, β2 and β5) of the constitutive (c) and immunoproteasome (i) was determined using Activity-Based Probes (ABP) labelling followed by SDS-PAGE electrophoresis. The gel images were acquired, the intensity of fluorescence for each subunit was determined and the activity ratio between immuno and constitutive proteasome for each proteasome β subunit in a cohort of BCP-ALL and T-ALL is presented.

Samples	BCP-ALL			T-ALL		
	β5i/c	β1i/c	β2i/c	β5i/c	β1i/c	β2i/c
Number of samples	28	28	28	21	21	21
Minimum	0.86	2.08	0.58	0.71	0.20	0.03
25% Percentile	3.57	4.91	0.94	1.42	0.85	0.28
Median	6.11	5.85	1.32	1.96	1.35	0.45
75% Percentile	10.31	11.53	1.93	3.34	2.22	0.64
Maximum	33.41	55.83	4.11	8.74	6.90	1.11
Mean	8.47	9.51	1.51	2.73	1.82	0.49
Std. Deviation	7.23	10.28	0.81	2.21	1.63	0.28
Std. Error	1.37	1.91	0.15	0.48	0.36	0.06
Lower 95% CI of mean	5.67	5.60	1.20	1.73	1.07	0.36
Upper 95% CI of mean	11.27	13.42	1.83	3.74	2.56	0.62
